# Conformity-like behaviour in mice observing the freezing of other mice: a model of empathy

**DOI:** 10.1186/s12868-020-00566-4

**Published:** 2020-05-01

**Authors:** Hiroshi Ueno, Shunsuke Suemitsu, Shinji Murakami, Naoya Kitamura, Kenta Wani, Yu Takahashi, Yosuke Matsumoto, Motoi Okamoto, Takeshi Ishihara

**Affiliations:** 1 Department of Medical Technology, Kawasaki University of Medical Welfare, 288, Matsushima, Kurashiki, Okayama 701-0193 Japan; 2 Department of Psychiatry, Kawasaki Medical School, Kurashiki, 701-0192 Japan; 3 Department of Neuropsychiatry, Graduate School of Medicine, Dentistry and Pharmaceutical Sciences, Okayama University, Okayama, 700-8558 Japan; 4 Department of Medical Technology, Graduate School of Health Sciences, Okayama University, Okayama, 700-8558 Japan

**Keywords:** Behaviour, Conformity, Emotional contagion, Empathy, Mouse, Imitation, Locomotion, Mimicry, Observational fear, Rodent, Social interest

## Abstract

**Background:**

Empathy refers to the ability to recognise and share emotions with others. Several research groups have recognised observational fear in mice as a useful behavioural model for assessing their ability to empathise. However, in these observation systems, it remains unclear whether the observer mouse truly recognises the movements of, and empathises with, the demonstrator mouse. We examined changes in the behaviour of an observer mouse when a demonstrator mouse was anaesthetised, when the demonstrator’s activity was increased, and when the interval of electrical stimulation was altered. If mice exhibit an ability to empathise, then the observer should display empathic behaviour when the demonstrator experiences pain or discomfort under any circumstances.

**Results:**

Relative to low-frequency stimulation, frequent electrical stimulation reduced immobility time among observer mice. Moreover, when demonstrators exhibited excessive activity, the activity of the observers significantly increased. In addition, the proportion of immobility time among observer mice significantly increased when demonstrator mice exhibited fear learning and excessive immobility.

**Conclusion:**

Although our results indicate that observer mice change their behaviour based on the movements of demonstrator mice, increases in immobility time may reflect conformity-like behaviour rather than emotional empathy. Thus, not only visual but also auditory and odour information additionally influenced the conformity-like behaviour shown by observer mice. Thus, our findings suggest that methods other than the fear observation system should be used to investigate rodent empathy-like behaviour.

## Background

Empathy, which refers to the ability to share and understand the emotions of others, is an important element of our social and emotional lives. Empathy is also crucial for emotional experiences and social interactions in social animals [1–3].

Impairments in empathy are a hallmark of many neuropsychiatric disorders, including autism spectrum disorder (ASD) and psychosis [4, 5]. In extreme cases, lack of empathy can lead to severe aggression against others [6, 7]. However, little is known regarding the neurobiological substrates underlying empathy. Appropriate animal models of empathy are thus required to more fully elucidate the neurobiological systems involved in empathy and to develop clinical treatment strategies for patients with low empathy.

Historically, empathy has been regarded as a high-level cognitive process unique to humans and primates. However, recent studies have reported that non-primates [1, 8, 9], birds [10, 11], and rodents [12, 13] exhibit empathy-like behaviour. Indeed, research has suggested that empathy for pain or distress is mediated by common neural and neuroendocrine processes in both humans and animals [2, 14–22]. Mice and rats may exhibit empathy-related behaviours such as observational fear [23–25], social coordination of pain [12], comfort [26], and prosocial support behaviour [27, 28]. Several research groups have recognised observational fear as a useful behavioural model for assessing their ability to empathise [2, 19, 20, 29, 30]. In the fear observation system, the observer mouse exhibits defensive immobility upon witnessing the distress of an allogeneic demonstrator mouse subjected to an electric shock [23, 24, 31]. This phenomenon, known as emotional state matching or influence sharing, has been regarded as a measure of socially transmitted fear [23, 32].

In the last decade, paradigms designed to investigate indirect fear conditioning in laboratory rodents have rapidly emerged in the literature [33]. However, in these observation systems, it remains unclear whether the observer mouse truly recognises the movements of and empathises with the demonstrator mouse. Given that even humans cannot know exactly what others feel and think, it is necessary to exercise caution when discussing whether mice and rats have an ability to empathise [34–36].

In the present study, we utilised the fear observation system to alter emotional and behavioural states in demonstrator mice to examine alterations in the behaviour of observer mice. If mice exhibit an ability to empathise, then the observer should display empathic behaviour when the demonstrator experiences pain or discomfort under any circumstances. However, our results indicate that observer behaviour in the fear observation system may reflect conformity-like or imitative behaviour rather than empathy-like behaviour.

## Results

### Observer behaviour following foot shock to cagemate mice or fear-conditioned cagemate mice

**Fig. 1:**
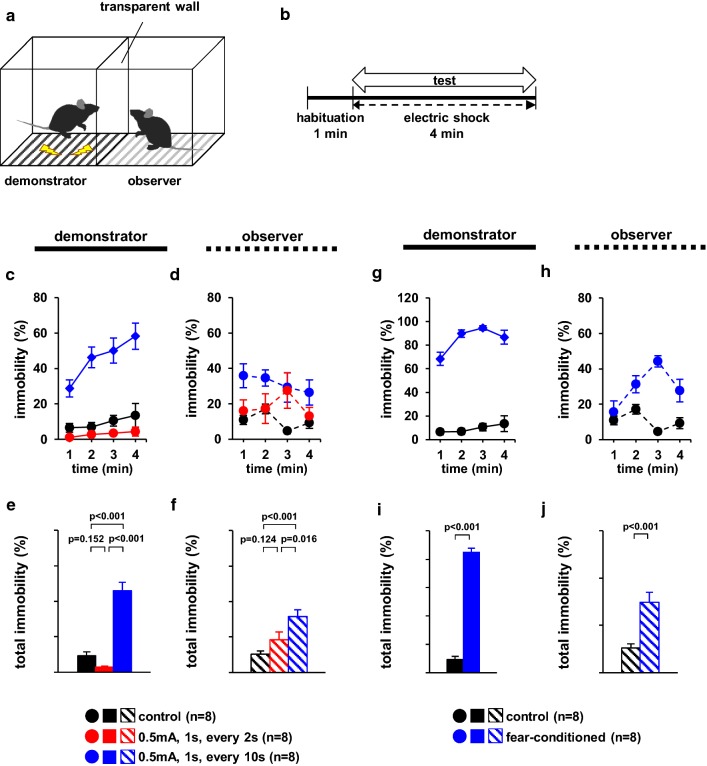
Observer behaviour toward foot-shocked or fear-conditioned cagemate mice. **a** Schematic diagram of the experiment. **b** Outline of the behavioural paradigm. Graphs showing the proportion of time spent immobile during each 1-min period (**c**, **d**, **g**, **h**) and the total time spent immobile (**e**, **f**, **i**, **h**). Left panels (**c**, **e**, **g**, **h**): demonstrators. Right panels (**d**, **f**, **h**, **j**): observers. All data are presented as the mean ± SEM. Statistical significance is represented by asterisks: *p < 0.05. The p-values were calculated using two-way repeated-measures analysis of variance (ANOVA) in **c**, **d**, **g,** and **h** and one-way ANOVA in **e**, **f**, **i,** and **f**. *SEM* standard error of the mean

In this experiment, we examined whether the observer mice exhibited different immobility times when the cagemate mice received electrical shocks at different stimulation frequencies (Additional files 1, 2, 3). To increase the degree of pain in the demonstrator mice, we selected a shock time of one every 2 s. Previous reports have argued that empathy-like behaviour in observer mice should be reflected by a significant increase in immobility time due to the increased pain in the demonstrator mice. Therefore, the demonstrator cagemate mice received no foot shock (control), a 1-s foot shock every 2 s, or a 1-s foot shock every 10 s for 4 min. During the test period, the percentage of time spent immobile was significantly higher in the demonstrator mice shocked every 10 s than in the other two groups (Fig. 1c, *F*_2,21_ = 120.394, p < 0.001; control vs. 2 s, p = 0.455; control vs. 10 s, p < 0.001; 2 s vs. 10 s, p < 0.001, Fig. 1e, *F*_2,20_ = 57.168, p < 0.001; control vs. 2 s, p = 0.152; control vs. 10 s, p < 0.001; 2 s vs. 10 s, p < 0.001). Among demonstrators, the percentage of time spent immobile was significantly lower in mice shocked every 2 s than in mice shocked every 10 s (Fig. 1c, e). Among the observers, the percentage of time spent immobile was significantly higher when viewing demonstrator mice that received a foot shock every 10 s than when viewing those that received no foot shock or a 1-s foot shock every 2 s (Fig. 1d, *F*_2,20_ = 101.390, p < 0.001; control vs. 2 s, p = 0.373; control vs. 10 s, p = 0.001; 2 s vs. 10 s, p = 0.048, Fig. 1f, *F*_2,20_ = 9.784, p = 0.011; control vs. 2 s, p = 0.124; control vs. 10 s, p < 0.001; 2 s vs. 10 s, p = 0.016). We observed no significant differences in the behaviour of the observer mice when demonstrator mice received no foot shock or a foot shock every 2 s (Fig. 1d, f).

Next, we examined whether the observer mice exhibited increased immobility time when viewing demonstrator mice with high immobility, relative to control demonstrators (Additional file 4). The results of the control mice (Fig. 1g–j) are based on the results of the control mice of Fig. 1c–f. During the test period, the percentage of time spent immobile was significantly higher in the fear-conditioned demonstrator mice than in the control mice (Fig. 1g, *F*_1,18_ = 549.467, p < 0.001, I, *t*_*18*_ = − 18.776, p < 0.001). Among observers, the percentage of time spent immobile was significantly higher when viewing fear-conditioned demonstrator mice than when viewing demonstrator mice that had not received a foot shock (Fig. 1h, *F*_1,18_ = 99.731, p < 0.001, Fig. 1j, *t*_*14*_ = − 4.772, p < 0.001).

### Observer behaviour following MK-801-induced hyperactivity or anaesthetisation of cagemate mice

**Fig. 2:**
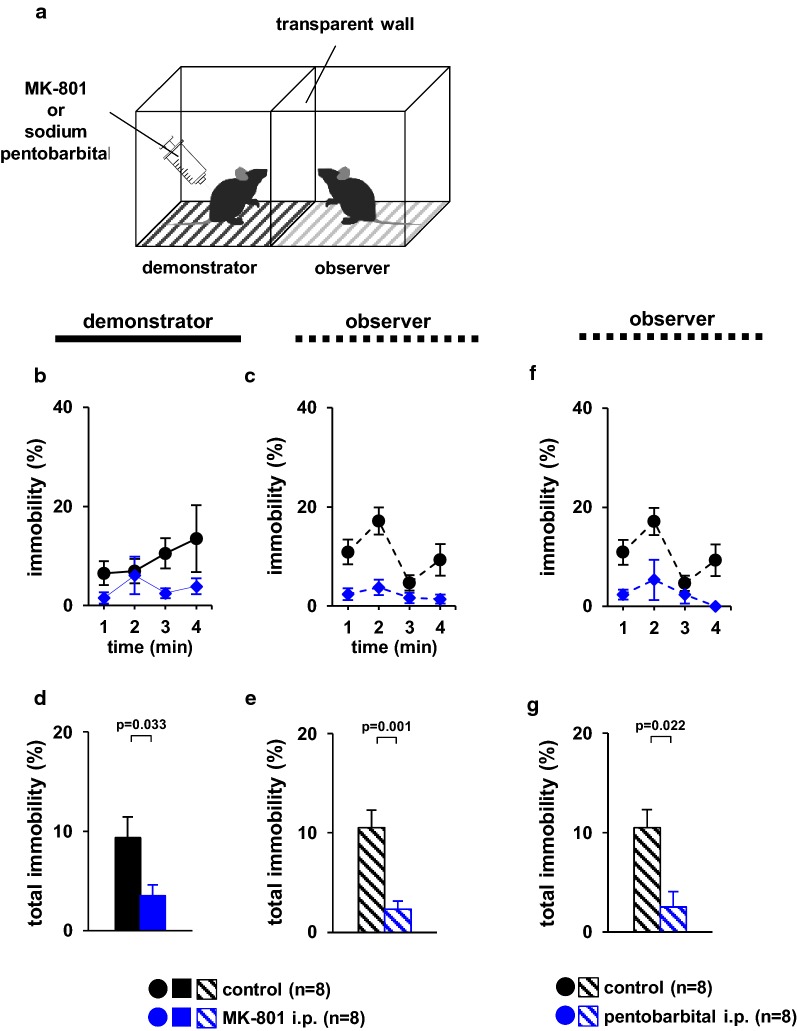
Observer behaviour toward cagemate mice with MK-801-induced hyperactivity or anaesthetised cagemate mice. **a** Schematic diagram of the experiment. **b** Outline of the behavioural paradigm. Graphs showing the proportion of time spent immobile during each 1-min period (**b**, **c**, **f**) and the total time spent immobile (**d**, **e**, **g**). Left panels (**b**, **d**): demonstrators. Right panels (**c**, **e**, **f**, **g**): observers. All data are presented as the mean ± SEM. Statistical significance is represented by asterisks: *p < 0.05. The p-values were calculated using two-way repeated-measures analysis of variance (ANOVA) in **b**, **c,** and **f** and one-way ANOVA in **d**, **e,** and **g**. *SEM* standard error of the mean

We investigated whether immobility times among observer mice decreased when demonstrator mice exhibited excessive activity due to MK-801 treatment (Additional file 5). During the test period, the percentage of time spent immobile was significantly lower in MK-801-treated demonstrator mice than in control mice (Fig. 2b, *F*_1,18_ = 27.280, p < 0.001, Fig. 2d, *t*_*14*_ = − 2.374, p = 0.034). Among observers, the percentage of time spent immobile was significantly lower when viewing MK-801-treated mice than when viewing demonstrator mice that had not received a foot shock (Fig. 2c, *F*_1,18_ = 41.570, p < 0.001, Fig. 2e, *t*_*14*_ = 4.130, p = 0.001).

We examined whether mice exhibited differences in immobility times when viewing anaesthetised demonstrator mice. Among observers, the percentage of time spent immobile was significantly lower when viewing anaesthetised mice than when viewing mice that had not received a foot shock (Fig. 2f, *F*_1,18_ = 26.104, p < 0.001, Fig. 2g, *t*_*14*_ = 2.573, p = 0.022).

### Locomotor activity in the presence of demonstrator cagemate mice

**Fig. 3:**
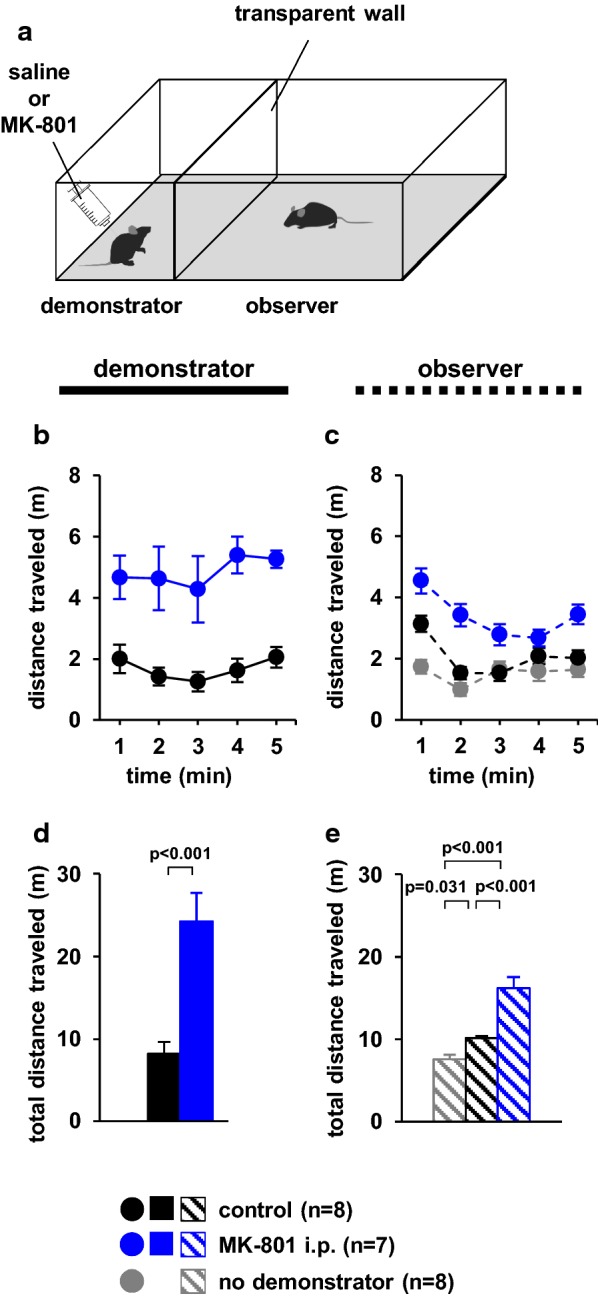
Locomotor activity in the presence of demonstrator cagemate mice. **a** Schematic diagram of the experiment. Graphs showing the distance travelled during each 1-min period (**b**, **c**) and the total distance travelled (**d**, **e**). Left panels (**b**, **d**): demonstrators. Right panels (**c**, **e**): observers. All data are presented as the mean ± SEM. Statistical significance is represented by asterisks: *p < 0.05. The p-values were calculated using two-way repeated-measures analysis of variance (ANOVA) in **b** and **c** and one-way ANOVA in **d** and **e**. *SEM* standard error of the mean

In this experiment, we examined whether observer mice exhibited differences in locomotor activity when viewing mice treated with or without MK-801 (Fig. 3a). During the test period, the distance travelled was significantly higher in MK-801-treated demonstrator mice than in control mice (Fig. 3b, *F*_1,14_ = 109.795, p < 0.001, Fig. 3d, *t*_*12*_ = − 5.174, p < 0.001). Among observers, the distance travelled was significantly higher when viewing untreated mice than when viewing mice in the absence of a demonstrator mouse (Fig. 3c, *F*_4,88_ = 3.911, p = 0.001; control vs. MK-801, p = 0.036; control vs. no demonstrator, p < 0.000; MK-801 vs. no demonstrator, p < 0.001, Fig. 3e, *F*_2,20_ = 28.737, p < 0.001; control vs. MK-801, p = 0.031; control vs. no demonstrator, p < 0.001; MK-801 vs. no demonstrator, p < 0.001). Observer mice travelled a significantly greater distance in the presence of MK-801-treated demonstrator mice than in the presence of control demonstrator mice (Fig. 3c, e).

### Observer behaviour following foot shock to cagemate or fear-conditioned cagemate mice in a chamber with an opaque divider

**Fig. 4:**
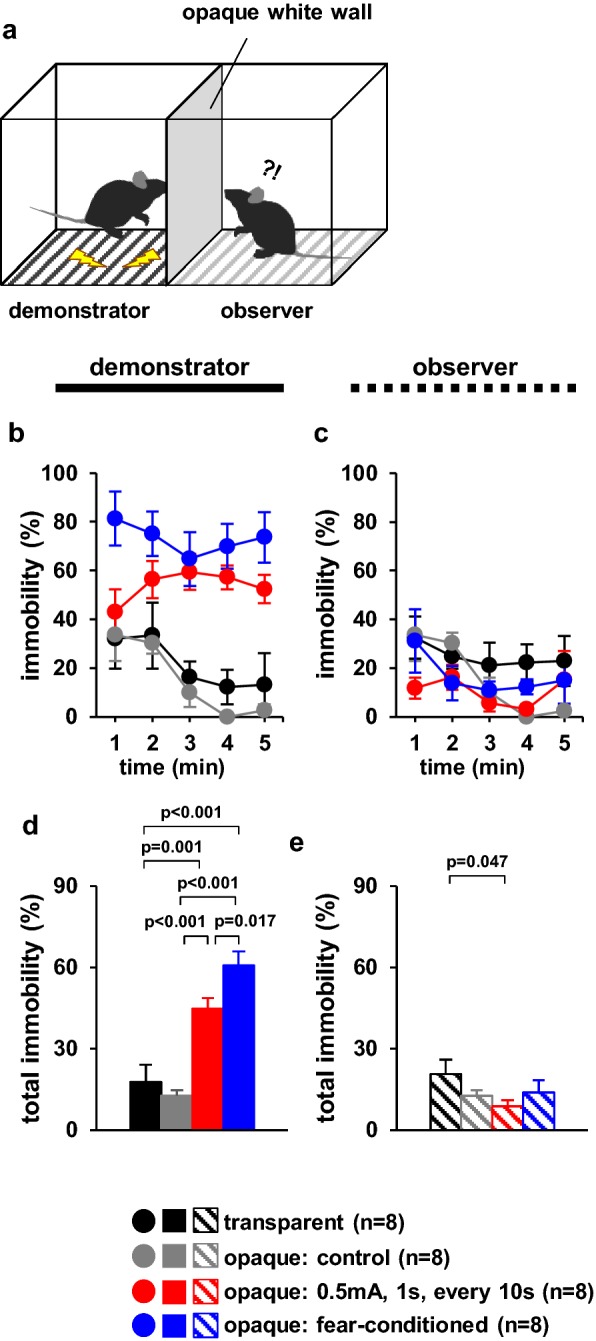
Observer behaviour toward foot-shocked or fear-conditioned cagemate mice in a chamber with an opaque divider. **a** Schematic diagram of the experiment. Graphs showing the proportion of time spent immobile during each 1-min period (**b**, **c**) and the total time spent immobile (**d**, **e**). Left panels (**b**, **d**): demonstrators. Right panels (**c**, **e**): observers. All data are presented as the mean ± SEM. Statistical significance is represented by asterisks: *p < 0.05. The p-values were calculated using two-way repeated-measures analysis of variance (ANOVA) in **b** and **c** and one-way ANOVA in **d** and **e**. *SEM* standard error of the mean

Observers may have obtained visual, auditory, and odour information from the demonstrator. We need to evaluate information that changes observer behaviour. To examine the ability of mice for visual discrimination, we used an opaque divider so that the observer could not see the demonstrator. In this experiment, we examined whether observer mice exhibited different immobility times when cagemate mice received electrical shocks beyond the opaque divider (Fig. 4a). However, this experimental device does not block both auditory and odour information. The demonstrator cagemate mice received no foot shock in a chamber with a transparent divider or an opaque divider (control) or a 1-s foot shock (0.5 mA) every 10 s for 5 min. We used fear-conditioned demonstrator cagemate mice.

During the test period, the percentage of time spent immobile was significantly higher in demonstrator mice shocked every 10 s than in the two groups that received no foot shock (Fig. 4b, *F*_4,64_ = 2.792, p = 0.033; transparent vs. control, p = 1.0; transparent vs. 10 s, p = 0.007; transparent vs. conditioned, p < 0.001; control vs. 10 s, p = 0.001; control vs. conditioned, p < 0.001; 10 s vs. conditioned, p = 0.103, Fig. 4d, *F*_3,28_ = 22.984, p < 0.001; transparent vs. control, p = 0.500; transparent vs. 10 s, p = 0.001; transparent vs. conditioned, p < 0.001; control vs. 10 s, p < 0.001; control vs. conditioned, p < 0.001; 10 s vs. conditioned, p = 0.017). Among the demonstrator mice, the percentage of time spent immobile was significantly higher in fear-conditioned mice than in mice shocked every 10 s (Fig. 4b, d).

Among observer mice, the percentage of time spent immobile was significantly lower when viewing demonstrator mice in a chamber with an opaque divider that received a foot shock every 10 s than when viewing those that received no foot shock in a chamber with an transparent divider (Fig. 4c, *F*_4,64_ = 1.134, p = 0.350; transparent vs. opaque control, p = 1.0; transparent vs. opaque 10 s, p = 0.284; transparent vs. opaque-conditioned, p = 1.0; opaque control vs. opaque 10 s, p = 1.0; opaque control vs. opaque-conditioned, p = 1.0; opaque 10 s vs. opaque-conditioned, p = 1.0; Fig. 4e, *F*_3,28_ = 1.553, p = 0.239; transparent vs. opaque control, p = 0.213; transparent vs. opaque 10 s, p = 0.047; transparent vs. opaque-conditioned, p = 0.243; opaque control vs. opaque 10 s, p = 0.477; opaque control vs. opaque-conditioned, p = 0.836; opaque 10 s vs. opaque-conditioned, p = 0.310). We observed no significant differences in the behaviour of observer mice when demonstrator mice received no foot shock or in fear-conditioned mice in a chamber with an opaque divider (Fig. 4c, e).

### Observer behaviour toward videos of foot-shocked cagemate mice

**Fig. 5:**
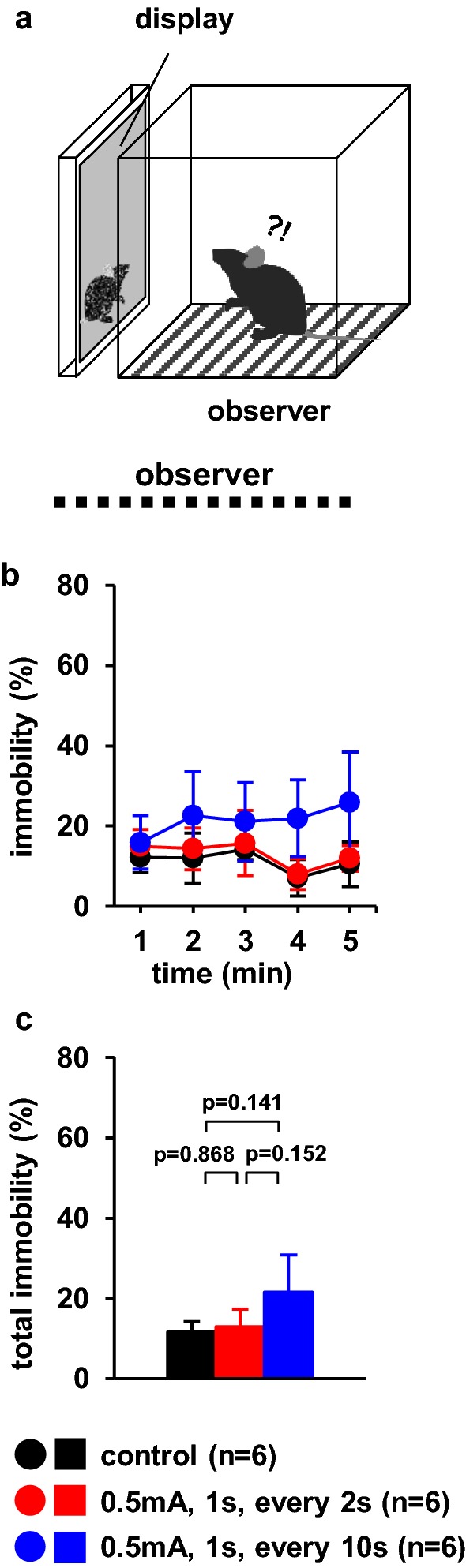
Observer behaviour toward videos of foot-shocked cagemate mice. **a** Schematic diagram of the experiment. Graphs showing the proportion of time spent immobile of observer during each 1-min period (**b**) and the total time spent immobile (**c**). All data are presented as the mean ± SEM. Statistical significance is represented by asterisks: *p < 0.05. The p-values were calculated using two-way repeated-measures analysis of variance (ANOVA) in **b** and one-way ANOVA in **c**. *SEM* standard error of the mean

We examined the immobility changes in response to observing videos of cagemate mice. We examined whether observer mice exhibited different immobility times when cagemate mice received electrical shocks in the display at different stimulation frequencies (Fig. 5a). We used videos where the demonstrator cagemate mice received no foot shock (control), a 1-s foot shock every 2 s, or a 1-s foot shock every 10 s for 5 min. Among observer mice, the percentage of time spent immobile was not significantly higher when viewing videos of demonstrator mice that received a foot shock every 10 s than when viewing videos of those that received no foot shock or a 1-s foot shock every 2 s (Fig. 5b, *F*_8,44_ = 1.098, p = 0.384; control vs. 2 s, p = 1.0; control vs. 10 s, p = 1.0; 2 s vs. 10 s, p = 1.0, Fig. 5c, *F*_2,15_ = 1.493, p = 0.260; control vs. 2 s, p = 0.868; control vs. 10 s, p = 0.141; 2 s vs. 10 s, p = 0.152). We observed no significant differences in the behaviour of observer mice when demonstrator mice received no foot shock or a foot shock every 2 s (Fig. 5b, c).

### Observer behaviour after electric shock toward foot-shocked cagemate mice

**Fig. 6:**
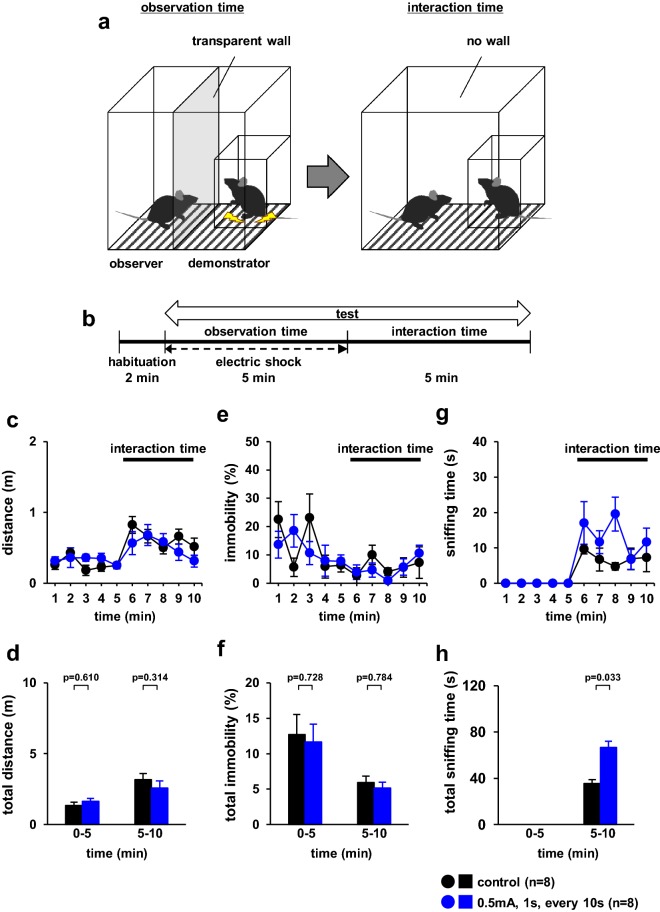
Observer behaviour after electric shock toward foot-shocked cagemate mice. **a** Schematic diagram of the experiment. **b** Outline of the behavioural paradigm. Graphs showing the distance travelled of observer during each 1-min period (**c**) and the total distance travelled (**f**). Graphs showing the proportion of time spent immobile of observer during each 1-min period (**e**) and the total time spent immobile (**f**). Graphs showing the time of sniffing the demonstrator cage during each 1-min period (**g**) and the total time of sniffing (**h**) in observer mice. All data are presented as the mean ± SEM. Statistical significance is represented by asterisks: *p < 0.05. The p-values were calculated using two-way repeated-measures analysis of variance (ANOVA) in **c**, **e**, and **g**, and one-way ANOVA in **d**, **f**, and **h**. *SEM* standard error of the mean

We investigated whether the observer showed empathy-like behaviour after observing the demonstrator that received electrical shocks. We examined whether the observer approached the demonstrator after observing the behaviour of the cagemate mice that received electrical shocks (Fig. 6a). There were no significant differences between the groups in the distance travelled (Fig. 6c, 0–5 min, *F*_4,32_ = 0.925, p = 0.462; 5–10 min, *F*_4,32_ = 1.532, p = 0.246; Fig. 6d, *F*_1,30_ = 1.214, p = 0.286; 0–5 min, p = 0.610; 5–10 min, p = 0.314). We observed no significant differences in the percentage of time spent immobile in observer mice when demonstrator mice received no foot shock or a foot shock every 20 s (Fig. 6e, 0–5 min, *F*_4,32_ = 2.203, p = 0.140; 5–10 min, *F*_4,32_ = 0.597, p = 0.667; Fig. 6f, *F*_1,30_ = 0.003, p = 0.958; 0–5 min, p = 0.728; 5–10 min, p = 0.784). During the post-opening period, the time spent sniffing the cage was significantly higher in electrically shocked demonstrator mice than in control mice (Fig. 6g, 5–10 min, *F*_4,28_ = 0.995, p = 0.427, Fig. 6h, 5–10 min, p = 0.033).

### Consumption of chocolate chips in the presence of foot-shocked demonstrator or observer cagemate mice

**Fig. 7:**
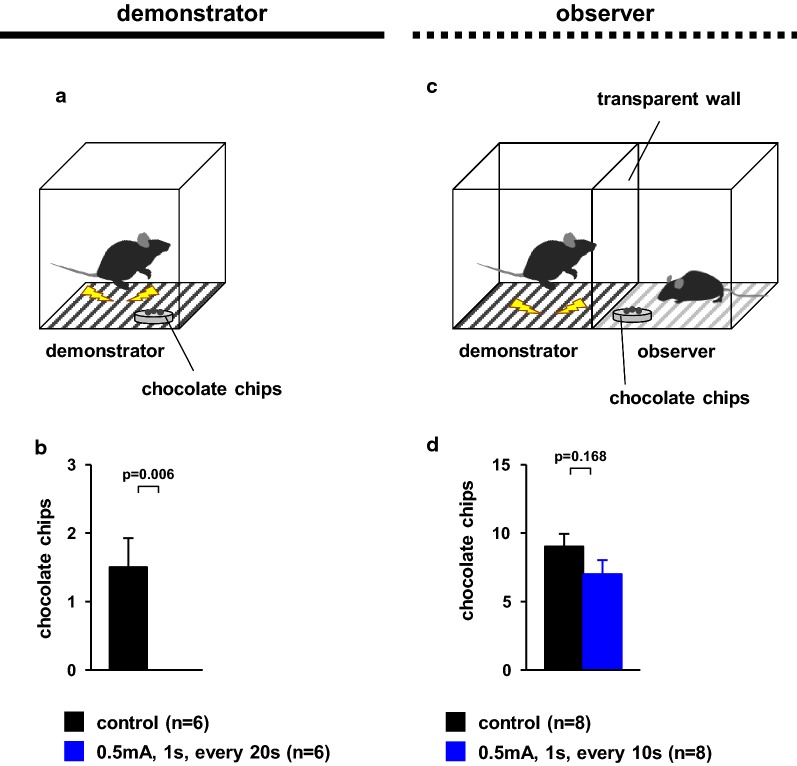
Consumption of chocolate chips in the presence of foot-shocked cagemate mice. **a**, **c** Schematic diagram of the experiment. Graphs showing the number of chocolate chips eaten by demonstrator mice (**b**) or observer mice (**d**). All data are presented as the mean ± SEM. Statistical significance is represented by asterisks: *p < 0.05. The p-values were calculated using t-tests (**b**, **d**)

To analyse if hunger trumps fear, we examined whether mice would eat chocolate chips in the presence of foot shock (Fig. 7a). During the test period, subject mice received a foot shock every 10 s. Mice ate chocolate chips when they did not receive foot shock. Subject mice that received a foot shock every 10 s did not eat chocolate chips (Fig. 7b, *t*_*14*_ = 2.323, p = 0.003).

Next, we examined whether observer mice would eat chocolate chips in the presence of foot-shocked demonstrator cagemate mice (Fig. 7c, Additional files 6, 7). During test period, demonstrator mice received a foot shock every 10 s. Although observer mice ate chocolate chips in the presence of foot-shocked cagemate mice (Additional file 1), there were no significant differences in the number of chocolate chips eaten between mice who viewed control demonstrators and those that viewed foot-shocked demonstrators (Fig. 7d, *t*_*14*_ = 1.45, p = 0.168).

## Discussion

In this study, we examined alterations in the behaviour of observer mice when viewing demonstrator mice subjected to various conditions in the fear observation system. Our findings indicated that, among observer mice, the proportion of immobility time—an index of empathy-like behaviour—changes based on the immobility time of the demonstrator mouse. These results show that mice visually recognise and tend to synchronise with the demonstrator’s movements. Such findings suggest that observer immobility in the fear observation system may reflect conformity-like or imitative behaviour rather than empathy-like behaviour.

Several previous studies utilising the fear observation system have used foot-shock intervals of 10 to 15 s to examine empathy-like behaviour in observer mice [23, 37–39]. However, it is unclear why these stimulation intervals were selected. In our study, when stimulation was provided every 2 s, the immobility time significantly decreased among demonstrators, while the degree of pain significantly increased relative to that observed when foot shocks were provided every 10 s. This reduction in immobility is due to the demonstrators escape attempts during the experiment, and therefore their mobility, upon receiving foot shocks every 2 s. Previous reports have argued that empathy-like behaviour in observer mice should be reflected by significant increases in immobility time due to increases in pain in demonstrator mice. However, the proportion of immobility time was lower among observer mice that viewed demonstrators receiving foot shocks every 2 s than among those viewing demonstrators receiving foot shocks every 10 s. Previous research have indicated that mice have the ability to visually recognise the behaviour of others [12]. In accordance with this finding, our results revealed that observer mice exhibited behavioural alterations based on the demonstrator’s movements at different stimulation frequencies. Thus, observers did not exhibit freezing or immobility (i.e. empathy-like behaviour) merely by viewing a demonstrator in distress. Although researchers have suggested that observers should become immobile when the demonstrator immobilises in preparation for the next foot shock, it remains unclear whether observers indeed empathised with demonstrators that received foot shocks every 2 s. Further studies are required to more fully understand the emotional states of observer mice. Consistent with these findings, we also noted significant increases in the percentage of immobility time among observer mice that viewed demonstrator mice with excessive immobility due to fear learning. This result is in accordance with previous findings regarding the fear observation system [33]. However, it remains unclear whether immobility among observer mice is due to empathy or conformity for the demonstrator.

MK-801 treatment significantly increased activity among demonstrator mice, leading to significant decreases in immobility time and increases in the distance travelled among observer mice, relative to levels observed in controls. Research has indicated that mice show an interest in cagemates exhibiting abnormal behaviour [40]. Furthermore, mice are social animals and tend to follow allogeneic individuals [41, 42], while rats tend to select the same feeding ground as conspecifics [43, 44]. Maintaining group cohesion is an important task for many animals. To maintain the population, each individual must be synchronised with regard to the direction and timing of movement. In this context, “conformity” refers to the tendency to imitate the movements of others and perform the same action. Interestingly, one previous study noted that rats paired with naïve partners in the fear observation system exhibit a reduced fear response in the conditioning context compared to animals paired with previously shocked partners [45]. Consistent with these findings, another study reported that demonstrators paired with naïve observers froze significantly less than demonstrators paired with shocked observers [24]. Taken together, these findings suggest that, in the fear observation system, observers may exhibit conformity-like or imitative behaviour rather than empathic behaviour.

Observer mice exhibited significant decreases in immobility time when viewing demonstrators that had been immobilised due to anaesthesia. This behaviour may not be a conformity-like behaviour. Previous studies have also indicated that mice show no interest in cagemates that are comatose due to anaesthesia [46]. The subject mouse may have interpreted the abnormal sleeping of their conspecific as a normal sleeping state. Mice can observe the movement of others and identify their emotional and behavioural states [40], and because mice rarely see stunned conspecifics in nature, they may not recognise it as abnormal. While our results indicated that observer mice did not imitate the movement of the demonstrator mouse under these conditions, changes in behaviour may indicate that they recognised the condition to some extent.

Mice can distinguish pictures using two-dimensional visual information [47]. Recent studies have shown that mice can recognise virtual reality spaces [48, 49]. However, in this study, the observer mouse did not change the immobility time even when viewing the video of the demonstrator mouse. Also, due to the lateral placement of the eyes, rodents have a wide field of view. Therefore, the flat screen may have caused a distortion in the rodent’s field of view [50, 51]. It is possible that the mouse did not recognise the cagemate on the display as genuine. In addition, due to blocking the visual information of the observer mouse, the immobility of the observer mouse did not synchronise with that of the demonstrator mouse. These results indicate that mice visually identify cagemate behaviour.

However, immobility in observer mice was reduced when separated by a non-transparent divider from demonstrator mice receiving foot shock. This suggests that mice receive cues other than visual from the demonstrator mouse. Mice change their behaviour by olfactory or auditory information [46]. Olfactory and tactile cues are more effective to mice than visual cues [52, 53]. Mice mostly use olfactory cues for personal identification rather than other sensory cues [54]. Olfactory cues have also been reported to be important for empathy-like behaviours [55]. Further studies are warranted to determine the implications of the reduced immobility observed in this study.

We have shown that observer mice are highly interested in demonstrator mice that have received electric shock. Mice have been reported to be interested in cagemates who behave abnormally [40]. In this study as well, demonstrator mice that received electric shock exhibited unusual behaviour. This result suggests that observer mice are highly interested in the abnormal behaviour of demonstrator mice.

It is suggested that visual information and olfactory information are important for mice to be interested in the abnormal behaviour of others [12, 46, 55]. Further research is needed to evaluate if auditory information is important.

In demonstrator mice, fear was a stronger motivator than hunger. While the demonstrator mouse received a foot shock, the observer mouse ate chocolate chips as if the demonstrator mouse had not been affected. Previous studies have reported that rats exhibit rescue-like behaviour under similar circumstances, by giving priority to releasing companions over eating chocolate chips due to empathy [27]. However, our results indicated that observer mice were more strongly motivated by chocolate chips than by the inaction of demonstrator mice, suggesting that immobility/freezing among observer mice does not reflect empathy-like behaviour.

Our findings indicated that observer mice may have exhibited conformity-like or imitative behaviour rather than empathy-like behaviour when cagemate mice were subjected to electric shocks. Rapid imitation is an automatic response (< 1 s) through which an individual imitates another’s expression [56]. Indeed, human beings often mimic the posture, gestures, and social cues of partners without being aware of the phenomenon (i.e., the “chameleon effect”) [57, 58]. Furthermore, individuals imitating behaviour tend to report increases in sharing, preferences, and empathy with interaction partners [56]. Rapid imitation facilitates communication exchange and coordination of various behaviours [59, 60] in both humans and non-human primates [61, 62]. Recently, these phenomena have also been observed in dogs and bears [63, 64]. While emotional transmission, empathy, and rapid imitation are distinct concepts, they may interact with one another as well [65]. For example, the transmission of certain emotions (i.e., empathy) can be mediated by imitation [56, 66].

Rapid imitation is based on automatic perception–behaviour coupling of sensorimotor information in the motor regions of the brain [66, 67]. The discovery of mirror neurons in the premotor cortex and parietal cortex of monkeys provides neurophysiological evidence for such coupling [68, 69], which may represent the neural basis of empathy [70, 71]. However, the perception–action mechanism (PAM) [72, 73] and the role of the Miller system [73] remain controversial.

Imitation depends on social bonds among friends, acquaintances, and strangers, respectively [63]. This order is consistent with the findings of previous reports suggesting that rodents exhibit empathic behaviour [16, 17, 22]. In addition, individuals with autism spectrum disorders exhibit reduced empathy, as well as difficulties with various imitation tasks [74].

It remains controversial whether rodents exhibit empathy in the fear observation system. While our results do not deny that mice exhibit empathy-like behaviour, they indicate that observer mice may synchronise with the movements of demonstrator mice and mimic their behaviour in the fear observation system. Thus, other experimental methods may be more appropriate for investigating the presence or absence of empathy-like behaviour in rats and mice. The experimental method of emulating observer imitation in rodents needs to be re-evaluated. Further studies are required to clarify whether mice and rats exhibit empathy-like behaviour or synchrony-like behaviour in the fear observation system.

## Methods

### Animals

All animal experiments were performed in accordance with ARRIVE (Animal Research: Reporting of In Vivo Experiments) (http://www.nc3rs.org.uk/arrive-guidelines) and the U.S. National Institutes of Health (NIH) Guide for the Care and Use of Laboratory Animals (NIH Publication No. 80-23, revised in 1996), and were approved by the Committee for Animal Experiments at Kawasaki Medical School Advanced Research Center. All efforts were made to minimise the number of animals used and their suffering. C57BL/6N male mice (age: 10 weeks) were purchased from Charles River Laboratories (Kanagawa, Japan) and housed in cages (five animals per cage) with food and water provided ad libitum under a 12-h light/dark cycle at 23–26 °C. We used male mice for these studies to eliminate the effects of the oestrous cycle in females. Four to five mice were housed together in each cage. All behavioural tests were conducted in behavioural testing rooms between 09:00 and 16:00 h during the light phase of the circadian cycle. After the tests, all equipment was cleaned with 70% ethanol and super hypochlorous water to prevent bias based on olfactory cues. Behavioural tests were performed according to the test order described below. In these tests, we used naïve mice (Table 1). The sample size calculator was not used in this study. Mice were randomly divided into two groups: one was the demonstrator and the other was the observer. All experiments used naïve observer and demonstrator mice that had never experienced foot shocks. Cagemate mice were used as demonstrator mice. To minimize animal distress, if a mouse that received foot shock became immobile during the experiment, the experiment would be stopped immediately. At the end of the study, the animals were sacrificed by CO_2_ inhalation. A gradual fill rate of 20% chamber volume per minute displacement was used for CO_2_ euthanasia.

**Table 1:** Test conditions

Test	Figures		No	
			Demonstrator	Observer
Observer behaviour when viewing cagemate mice subjected to foot shock or fear-conditioned cagemate mice in a chamber with a transparent divider	Fig. 1	No foot shock (control)	8	8
		0.5 mA, 1 s, every 2 s	8	8
		0.5 mA, 1 s, every 10 s	8	8
		Fear-conditioned	8	8
Observer behaviour when viewing cagemate mice with MK-801-induced hyperactivity or anaesthetisation of cagemate mice	Fig. 2	MK-801	8	8
Locomotor activity in the presence of demonstrator mice	Fig. 3	Control	8	8
		MK-801	7	7
		No demonstrator		8
Observer behaviour when viewing cagemate mice subjected to foot shock or fear-conditioned cagemate mice in a chamber with an opaque divider	Fig. 4	Opaque: no foot shock (control)	8	8
		Opaque: 0.5 mA, 1 s, every 10 s	8	8
		Opaque: fear-conditioned	8	8
Observer behaviour when viewing cagemate mice subjected to foot shock in the display	Fig. 5	No foot shock (control)	–	6
		0.5 mA, 1 s, every 2 s	–	6
		0.5 mA, 1 s, every 10 s	–	6
Observer behaviour after viewing electric shock to foot-shocked cagemate mice	Fig. 6	No foot shock (control)	8	8
		0.5 mA, 1 s, every 10 s	8	8
Consumption of chocolate chips in the presence of foot-shocked cagemate mice	Fig. 7	Control	6	–
		0.5 mA, 1 s, every 20 s	6	–
		Control	8	8
		0.5 mA, 1 s, every 10 s	8	s8

### Drugs

Dizocilpine hydrogen maleate ((+)-MK-801; 130-17381) was purchased from FUJIFILM Wako Pure Chemical Corporation (Osaka, Japan) and sodium pentobarbital (Somnopentyl^®^) was purchased from Kyoritsu Seiyaku Corp., (Tokyo, Japan). Pentobarbital is a short-acting barbiturate. All reagents were diluted in saline and administered intraperitoneally. (+)-MK-801 was diluted to a concentration of 0.1 mg/mL and administered at a dose of 0.2 mg/kg. Cagemate mice were deeply anaesthetised via a high dose of sodium pentobarbital (50 mg/kg, IP).

### Observer behaviour when viewing cagemate mice subjected to foot shock or fear-conditioned cagemate mice in a chamber with a transparent divider

We have used different stimulation frequencies to evaluate whether the observer’s behaviour varied mainly due to the demonstrator mice activity. In this test, we examined whether observer mice exhibited different immobility times when cagemate mice received electrical shocks at different stimulation frequencies. The apparatus consisted of two identical chambers (18 cm × 20 cm × 40 cm) with a transparent divider in the middle and a metal grid floor connected to a shock scrambler. In the observer chamber, the electrical cord was removed, and no electric shock was administered (Fig. 1a). One mouse was placed in each chamber for 1 min, following which the demonstrator mouse received either (1) no foot shock (control), (2) a 1-s foot shock (0.5 mA) every 2 s for 4 min, or (3) a 1-s foot shock (0.5 mA) every 10 s for 4 min (Fig. 1b). We used the same apparatus to analyse observer’s behaviour in response to fear-conditioned cagemate mice. To ensure the mice had learned to fear electric shocks, they were placed in the appropriate chamber, where they received a 1-s foot shock (0.5 mA) every 10 s for 4 min. A fear-conditioned mouse was placed in the demonstrator chamber, while a cagemate mouse was placed in the observer chamber. The resultant behaviour was recorded for 5 min. Images were captured using a video camera, and immobility time was measured. The “immobility time” was defined as the period of at least 1 s without moving. Data were recorded and analysed using video tracking software (ANY-MAZE, Stoelting Co., Wood Dale, IL, USA).

### Observer behaviour when viewing cagemate mice with MK-801-induced hyperactivity or anaesthetisation of cagemate mice

To evaluate whether the observer’s behaviour mainly vary because of the demonstrator mice’s hyperactivity or immobility we have used drugs for demonstrators. We investigated whether immobility times among observer mice decreased when demonstrator mice exhibited excessive activity. We treated the demonstrator mice, with MK-801 to reduce the immobility. In the present study, (+)-MK-801 (dizocilpine hydrogen maleate; 130-17381, FUJIFILM Wako Pure Chemical Corporation) was diluted in saline at a concentration of 0.1 mg/ml. Mice received an intraperitoneal (IP) injection of MK-801 of 0.2 mg/kg 30 min before being tested. The demonstrator mice were treated with pentobarbital to induce sleep and increase their immobility. Cagemate mice were deeply anaesthetised via a high dose of sodium pentobarbital (50 mg/kg, IP; Somnopentyl^®^, Kyoritsu Seiyaku Corp. Tokyo, Japan). The experimental apparatus consisted of two identical chambers (18 cm × 20 cm × 40 cm) with a transparent divider in the middle and a metal grid floor. Treated mice were placed in the demonstrator chamber, while cagemate mice were placed in the observer chamber (Fig. 2a). The resultant behaviour was recorded for 4 min. Images were captured using a video camera, and immobility time was measured and evaluated using the ANY-MAZE software.

### Locomotor activity in the presence of demonstrator mice

We increased the amount of activity in the demonstrator mice to evaluate whether the observer’s activity was mainly provoked by the demonstrator’s activity. We examined whether the observer mice exhibited differences in locomotor activity when observing mice treated with or without MK-801. The cagemate demonstrator and observer mice were individually placed in the appropriate chambers, which were partitioned by a transparent divider. The demonstrator mouse was placed in the centre of one chamber, which was a square surface surrounded by high walls (10 cm × 60 cm × 40 cm). The observer mouse was placed in the centre of the other chamber, which was also a square surface surrounded by high walls (20 cm × 60 cm × 40 cm, Fig. 3a). During the test, the total distance travelled (m) was recorded. We first analysed the behaviour of an observer mouse (n = 8) in the absence of a demonstrator mouse, following which we analysed the observer’s behaviour in the presence of a demonstrator mouse. We then analysed the observer’s behaviour (n = 7) in the presence of a demonstrator mouse injected with MK-801 30 min before testing. The test chamber was illuminated at 100 lx. Data were collected over a 5-min period. Data were analysed using ANY-MAZE software.

### Observer behaviour when viewing cagemate mice subjected to foot shock or fear-conditioned cagemate mice in a chamber with an opaque divider

We investigated whether observer mice exhibit different immobility times when visual information from demonstrator mice was blocked. We used an opaque divider to evaluate whether the observer’s behaviour was mainly provoked by visual cues. The apparatus consisted of two identical chambers (20 cm × 20 cm × 20 cm) with an opaque white divider in the middle and a metal grid floor connected to a shock scrambler (Fig. 4a). In this device, auditory and odour information are not blocked. In the observer chamber, the electrical cord was removed, and thereby no electric shock was administered. One mouse was placed in each chamber for 1 min, following which the demonstrator mouse received either (1) no foot shock (transparent divider), (2) no foot shock (opaque divider: control), or (3) a 1-s foot shock (0.5 mA) every 10 s for 5 min. We used the same apparatus to analyse observer behaviour in response to fear-conditioned cagemate mice. The resultant behaviour was recorded for 6 min. Images were captured using a video camera, and immobility time was measured. Immobility time was evaluated using ANY-MAZE software.

### Observer behaviour when viewing cagemate mice subjected to foot shock in the display

We investigated whether the observer mice exhibit different immobility times when only visual and auditory information were received. We have used a display instead of the demonstrator to evaluate whether the observer’s behaviour was mainly provoked by only visual and auditory cues. The apparatus consisted of an identical chamber (20 cm × 20 cm × 20 cm) with a metal grid floor connected to a shock scrambler. In this chamber, the electrical cord was removed, and no electric shock was administered. One mouse was placed in this chamber for 1 min, following which the demonstrator mouse either received (1) no foot shock (control), (2) a 1-s foot shock (0.5 mA) every 2 s, or (3) a 1-s foot shock (0.5 mA) every 10 s in the display (HP Spectre × 360 Convertible Model 13-ae016TU, Fig. 5a) for 5 min. We showed a video in which a demonstrator mouse received a foot shock or no foot shock. The sound was switched on, and the display was kept at 1 cm away from the chamber. The resultant behaviour was recorded for 6 min. Images were captured using a video camera, and immobility time was measured. Immobility time was evaluated using ANY-MAZE software.

### Observer behaviour after viewing electric shock to foot-shocked cagemate mice

We investigated whether the observer mice showed empathy-like behaviour after observing the demonstrator mice receiving electrical shocks. We have removed a transparent divider after the foot shock to evaluate whether the observer mice showed empathy-like behaviour after observing the demonstrator mice receiving electrical shocks. The apparatus consisted of two identical chambers (20 cm × 20 cm × 20 cm) with a transparent divider in the middle and a metal grid floor connected to a shock scrambler. In the observer chamber, the electrical cord was removed and no electric shock was administered. A transparent cage (7.5 × 7.5 × 10 cm) with several holes (of 1 cm diameter) was placed at the end of the chamber in the demonstrator chamber (Fig. 6a). A demonstrator mouse was placed in the cage. One observer mouse was placed in the observer chamber for 1 min, following which the demonstrator mouse received no foot shock (control) or a 1-s foot shock (0.5 mA) every 10 s for 5 min and could freely explore in the observer chamber. After 5 min, the transparent divider in the middle was removed. The observer mouse could explore the entire chamber for the next 5 min. One side of the entire chamber was identified as the demonstrator area and the other side as the observer area. The resultant behaviour of the subject mouse was recorded for 10 min. Images were captured using a video camera. During the 10-min session we measured the distance travelled, immobility time, and time sniffing of the demonstrator cage (nose in 2 cm around the cage). Data were analysed using ANY-MAZE software.

### Consumption of chocolate chips in the presence of foot-shocked cagemate mice

We examined whether observer mice prioritised empathy-like behaviour over consumption of chocolate chips when cagemate mice received electrical shocks. To induce hunger, we restricted access to food on the day before testing. The apparatus consisted of one chamber (20 cm × 20 cm × 20 cm) and a metal grid floor connected to a shock scrambler (Fig. 7a). One mouse was placed in the chamber for 1 min, following which the demonstrator mouse received no shock (control) or a 1-s foot shock (0.5 mA) every 20 s for 4 min. Simultaneously, the subject mouse received 12 chocolate chips. All behaviours were recorded using a video camera along with the quantity of chocolate chips eaten by the subject mice during the 4-min session.

Next, the cagemate demonstrator and observer mice were individually placed in the appropriate chambers partitioned by a transparent divider. The apparatus consisted of two identical chambers (18 cm × 20 cm × 40 cm) with a transparent divider in the middle and a metal grid floor connected to a shock scrambler. In the observer chamber, the electrical cord was removed, and no electric shock was administered. One mouse was placed in each chamber for 1 min, following which the demonstrator mouse received no foot shock (control) or a 1-s foot shock (0.5 mA) every 10 s for 4 min, while the observer mouse received 12 chocolate chips (Fig. 7c). The demonstrator mice received no chocolate chips. All behaviours were recorded using a video camera, and the number of chocolate chips eaten during the 5-min session was recorded.

### Statistical analyses

Statistical analyses were performed using SPSS software (IBM Corp, Tokyo, Japan). Data were analysed using one-way analyses of variance (ANOVA) followed by Tukey’s test, two-way repeated-measures ANOVA followed by Fisher’s least significant difference (LSD) test, or Student’s t-tests. Differences with a *p* value < 0.05 were regarded as statistically significant. All data are presented as the mean ± the standard error of the mean (SEM).

## Conclusion

Our findings indicate that observer behaviour in the fear observation system may reflect conformity-like or imitative behaviour rather than empathy-like behaviour. These results highlight the need to establish a new experimental method for investigating the presence or absence of empathy-like behaviour in rodents.

## Supplementary information

**Additional file 1: Video 1.** Observer behaviour when viewing cagemate mice subjected to no foot shock (control) for 4 min. It is a 4 × speed movie.

**Additional file 2: Video 2.** Observer behaviour when viewing cagemate mice subjected to a 1-s foot shock every 10 s for 4 min. It is a 4 × speed movie.

**Additional file 3: Video 3.** Observer behaviour when viewing cagemate mice subjected to a 1-s foot shock every 2 s for 4 min. It is a 4 × speed movie.

**Additional file 4: Video 4.** Observer behaviour in response to fear-conditioned cagemate mice for 4 min. It is a 4 × speed movie.

**Additional file 5: Video 5.** Observer behaviour when viewing cagemate mice with MK-801-induced hyperactivity for 4 min. It is a 4 × speed movie.

**Additional file 6: Video 6.** Consumption of chocolate chips in the presence of no foot-shocked cagemate mice for 4 min. It is a 4 × speed movie.

**Additional file 7: Video 7.** Consumption of chocolate chips in the presence of foot-shocked cagemate mice for 4 min. It is a 4 × speed movie.

## Data Availability

Dataset available on reasonable request from the corresponding author.

## Abbreviations

ASD: Autism spectrum disorder
PAM: Perception–action mechanism
ANOVA: Analyses of variance
